# Use of Family Care Indicators and Their Relationship with Child Development in Bangladesh

**DOI:** 10.3329/jhpn.v28i1.4520

**Published:** 2010-02

**Authors:** Jena D. Hamadani, Fahmida Tofail, Afroza Hilaly, Syed N. Huda, Patrice Engle, Sally M. Grantham-McGregor

**Affiliations:** ^1^ ICDDR,B, GPO Box 128, Dhaka 1000, Bangladesh; ^2^ Institute of Nutrition and Food Science, University of Dhaka, Dhaka 1000, Bangladesh; ^3^ Cal Poly State University, San Luis Obispo, California, USA; ^4^ Centre for International Health and Development, Institute of Child Health, University College London, London, United Kingdom

**Keywords:** Care-giving behaviours, Child development, Cognitive development, Family care indicators, Home environment, Bangladesh

## Abstract

Poor stimulation in the home is one of the main factors affecting the development of children living in poverty. The family care indicators (FCIs) were developed to measure home stimulation in large populations and were derived from the Home Observations for Measurement of the Environment (HOME). The FCIs were piloted with 801 rural Bangladeshi mothers of children aged 18 months. Five subscales were created: ‘play activities’ (PA), ‘varieties of play materials’ (VP), ‘sources of play materials’, ‘household books’, and ‘magazines and newspapers’ (MN). All subscales had acceptable short-term reliability. Mental and motor development of the children was assessed on the Bayley Scales of Infant Development and their language expression and comprehension by mothers’ report. After controlling for socioeconomic variables, VP and PA independently predicted four and three of the developmental outcomes respectively, and MN predicted both the Bayley scores. The FCI is promising as a survey-based indicator of the quality of children's home environment.

## INTRODUCTION

An estimated 219 million children in developing countries are failing to fulfil their developmental potential in the first five years of life due to poverty, poor health, and malnutrition (1). Poor stimulation in the home is one of the main mechanisms through which poverty detrimentally affects child development (2–3). However, specific risk factors in the home environment that affect cognitive and socio-emotional development of children are not well-documented in developing countries, and there are no globally-agreed indicators that could be easily assessed.

The dimensions of the home environment usually assessed are the quality of stimulation and learning opportunities, and the most commonly-used and validated instrument across countries is the Home Observations for Measurement of the Environment (HOME) (4–5). It provides a reasonably broad coverage of the social and physical conditions considered to influence both cognitive and socio-emotional development and is associated with child development in both developed (6–7) and developing countries (8–15). In a review of studies using the HOME from Africa, Asia, and Latin America, Bradley and Corwyn concluded that, although there are large differences in parenting across cultural groups, there remains significant variation in patterns within cultures that is associated with child competence (2).

Although the HOME is a good measure of the home environment, the scale is not suitable for use in large-scale population surveys. The HOME takes 45–60 minutes to administer and requires skilled, well-trained interviewers and considerable adaptation when used in developing countries. Moreover, the HOME involves observations, which are more difficult to standardize. Therefore, there is an urgent need for indicators that are simple, easy to administer, and applicable across different cultures for use in large population surveys. Such an instrument could contribute to assessing the size of the problem of poor home stimulation and monitoring interventions.

United Nations Children's Fund (UNICEF) developed the family care indicators (FCIs) questionnaire to measure the home environment of young children in developing countries in large population surveys, emphasizing items likely to be related to cognitive and language development. Items were adapted from several sources, including the HOME. Dimensions of the HOME measured here were derived from the Learning Materials, Parental Involvement, and Variety of Experiences subscales of the Infant Toddler version of the inventory (16).

Large household surveys that measure other aspects of children's well-being provide an opportunity for collecting information on the family environment. We proposed the addition of the FCI questionnaire to the Multiple Indicator Cluster Survey, which the UNICEF conducts in a number of developing countries every 3–5 years. Therefore, the questionnaire needed to meet the following criteria: be easily administered by trained but not specialized surveyors, contain a relatively few questions, be clear enough to be used for advocacy purposes, and be valid within and across cultures.

Determining the validity of the FCIs across cultures requires studies in a number of cultural settings and is beyond the scope of this study. The specific aims of the present study were to: (a) assess the test-retest reliability and stability over time of the FCI subscales; (b) examine their relationship with concurrent measures of children's development at 18 months, the HOME, and socioeconomic background; and (c) identify a subset of items for use in household surveys in developing countries.

## MATERIALS AND METHODS

International Centre for Diarrhoeal Disease Research, Bangladesh (ICDDR,B) conducted a large longitudinal study to examine the effects of giving food and micronutrient supplementation to pregnant women on birth outcomes. The study was conducted in Matlab, a rural area in the east-central plain of Bangladesh. The sample was recruited through regular surveys of the homes when all newly-pregnant women were enrolled. To determine the effect of supplementation on the developmental outcomes of the offspring, a subsample of 2,116 children born to these women during May 2002–Decemeber 2003 was assessed at seven (17) and 18 months of age. We took the opportunity of the child development component of the study to pilot the FCIs in a subsample and assess the relationship of the FCIs with measurements collected as part of the larger study.

### Sample

The present study was conducted over a seven-month period from January to July 2004, and all available mothers of children aged 18 months in the main study during this time were given the FCI (n=801) at home. Additionally, we interviewed 129 mothers of children who reached their first birthday during the initial months of the study and re-interviewed them when their children were aged 18 months to examine the stability of the FCI over this time period.

### Measurements

We used the following measures of social background, the HOME, child development and growth collected in the main study.

***Social background*:** On enrollment, i.e. at the beginning of pregnancy, information on age of the mother, parental education and occupation, and structure of the house was collected. The housing was categorized as 0=any part made from mud and 1=made from other materials. Income and expenditure ratio was assessed and coded as in deficit or in balance. In addition, the possession of certain household items (e.g. television, radio, domestic animal, chair, table, bed, bicycle, and rickshaw) was recorded and the items were then factor-analyzed, and the resulting factor was used as a wealth index (18). Weights of children were measured in the home within 72 hours of birth, their lengths and weights were measured at 18 months of age, and weight-for-age (WAZ), weight-for-height (WHZ), and height-for-age (HAZ) scores were calculated.

***Home stimulation*:** At the same home-visit when the FCI questionnaire was given, the mothers were also given a modified version of Caldwell's HOME (4) to measure the quality of stimulation in the home. One of the four research assistants interviewed them. Before starting the study, the interviewers were trained and reliabilities with the trainer assessed. Each interviewer conducted five interviews with non-study mothers and children and observed and scored 15 other interviews in presence of the trainer to assess inter-observer reliability. The intraclass correlation for each of the four interviewers with the trainer ranged from r=0.94 to 0.99 (n=20).

### Children's development

***Mental and psychomotor development*:** At 18 months of age, the children's development was assessed with the Revised Version of Bayley Scales of Infant Development (BSID-II) (19) using its Mental and Psychomotor Development Indices (MDI and PDI). The children were tested in the presence of their mothers at one of the four local health centres. The Bayley Scales have not been standardized for Bangladeshi children but have been used by the same research group in several previous studies in rural (20) and urban (9, 21–22) Bangladeshi children. The children's scores were in the normal range and correlated with parental education, socioeconomic status, and HOME scores in a theoretically-sensible way. Five psychologists were trained to test the children, and before beginning the study, each of them performed 10 tests on non-study children of the similar age range and was observed by a trainer. The intraclass correlations between the trainer and each psychologist ranged from r=0.88 to 0.99 (n=10) for both MDI and PDI.

***Language*:** The children's comprehensive and expressive language development was assessed at 18 months of age using an inventory, specially developed for Bangladesh, based on the principles of the MacArthur Communicative Development Inventory: words and gestures (23–24). The inventory depends on mothers’ report of their children's ability to comprehend and express words, arranged in categories (e.g. animals, body-parts, and food). There is a short version of 89 words that contains only nouns, verbs, and sounds but no gestures (25). The Bangladeshi inventory contained 60 words arranged in the same categories in order of difficulty (Hamadani JD *et al*. Personal communication, 2010). The inventory was developed after extensive piloting with mothers of young children and in consultation with Larry Fenson (Personal communication, 2003) and was then given to mothers in their homes. The test-retest reliabilities after 7–14 days in 15 mothers of children aged 18 months for comprehension and expression were (intraclass correlation) r=0.67 and 0.99 respectively.

***Family care indicators*:** The FCI questionnaire was developed by groups of experts organized by the UNICEF with preliminary piloting for comprehension in several countries (26). The items were grouped into the following theoretical subgroups: ‘Varieties of play materials’ (including picture books for young children) (7 items), which classified toys by their use; ‘Sources of play materials’ (4 items), which identified where the play materials came from; and ‘Play activities’ (6 items), which identified specific types of activities done by any adult in the home with the child in the previous three days. All these items were scored: yes=1 and no=0 (presence or absence of play material or activity). Two other items—‘Household books’, i.e. the number of books in the home, excluding picture books for young children (1 item) and ‘Magazines’, i.e. the number of magazines and newspapers in the home (1 item)—were initially intended to make one subscale; however, they behaved differently in the analyses, and we decided to keep them separate.

The FCI inventory was given in the children's homes by one of four research assistants. The interviewer asked to see items concerning play materials and reading materials whereas responses to the remaining items depended on mothers’ report. Before beginning the study, each interviewer conducted five interviews and observed and scored 15 more in the presence of the trainer, and intraclass correlation for each interviewer was 0.99.

### Statistics

We used the SPSS software (Windows version 12) (SPSS Inc, Chicago) for analyzing data. Frequency distributions of all the items on the FCI questionnaire were determined, and the FCI items in each of the subscales were summed to make totals. The data were examined for normality. Language expression was skewed and was normalized by log transformation. We assessed short-term test-retest reliability of the FCI over 7 to 14 days and longer-term stability from 12 to 18 months with intraclass correlations.

Both language scores, MDI, and PDI correlated with age, and age was, therefore, controlled in all analyses involving developmental measures. Language expression significantly correlated with sex (r=0.15, p<0.001), with girls producing more words than boys. Sex was, therefore, controlled in the multiple regression analyses of language expression. Correlations of the FCI subscales were calculated with all measures of socioeconomic background, childbirth characteristics, and concurrent developmental measures controlling for age. To determine if the FCI scales predicted development independent of socioeconomic variables, multiple regression analyses of each developmental measure were computed, entering age in the first step, then offering socioeconomic variables and any child characteristics that significantly correlated with both outcome measures and FCI scales in univariate analyses in the second step and then offering the FCI subscales in the last step.

### The most parsimonious FCI scale

To obtain the most parsimonious scale of stimulation in the home for Bangladesh, we first repeated multiple regression analyses of the four developmental outcomes offering the individual questions instead of the scales. The following questions independently predicted at least one of the Bayley and language scores (data not shown): sing songs, tell stories, read to child, take out of home, possesses toys bought from store, toys that make music, things for drawing and writing and toys for pretending, and presence of magazines and newspapers in the home. These questions were then summed to make a single FCI scale, and we examined whether this scale independently predicted the child development by repeating the four multiple regressions but offering only the single FCI scale.

To explore whether the association between the FCI subscales or single FCI scale and child development was linear or if there were thresholds below which child development was affected, we examined the difference in the MDI scores by the number of ‘play activities’, ‘varieties of play materials’, and ‘single FCI scale’ using analyses of co-variance (ANOCOVA) controlling for age.

### Ethics

Written consent was obtained from the guardians of the children. The research and ethical review committees of ICDDR,B approved the project.

## RESULTS

### Characteristics of the sample

Mothers of 801 children aged approximately 18 months were given the FCI, and 129 of them had earlier received the FCI when their children were aged 12 months. At 18 months of age, all children were assessed on the Bayley Scales, 788 also had their language assessed, and 797 were given the HOME (Table 1). Missing data on language assessment were due to delay in the development of the test. Anthropometric data were not available for 61 children, since they were not at home when the anthropometrists visited. Almost 50% of the mothers had either no formal education or had not completed five years of schooling, and only 11.7% had completed secondary school education (10 years of formal education). Fathers had slightly better educational levels than mothers (chi-square p<0.001). There were equal numbers of boys and girls in the sample.

**Table 1.** Characteristics of study population (n=801)

Variable	Value (mean±SD/%)
Age (months)	18.3±0.6
Education (years) of mothers (n=789)	
<5	50.4
5–9	37.9
≥10	11.7
Education (years) of fathers (n=781)	
<5	47.4
5–9	33.4
≥10	19.2
House made with some mud (n=789)	25
Occasional or constant income/expenditure deficit	18.6
Number of siblings	
None	31
1–2	55
3 or higher	14
Gestational age (weeks)	39.2±1.7
Birthweight (g)	2,688.7±403.4
Child measures at 18 months of age	
Bayley MDI	77.3±12.4
Bayley PDI	93.9±15.7
Language comprehension (n=788)	36.3±7.1
Language expression (median, interquartile range) (n=788)	10.5±7.1 (9.0, 6–13)
Total HOME (n=797)	29.7±6.7
Height-for-age z-score (n=739)	−1.95±1.08
Weight-for-age z-score (n=740)	−1.63±1.05
Weight-for-height z-score (n=740)	−0.94±0.99

HOME=Home Observations for Measurement of the Environment;

MDI=Mental development index;

PDI=Psychomotor development index;

SD=Standard deviation

### Frequency distribution of FCI subscales

The frequency of responses to each item of the FCI questionnaire and the subscale totals are shown in Table 2. A few items lacked variation: most children played with household objects (98%) and things from outside (99.3%), and nearly all children were taken outside the home (93.8%) whereas toys for stacking or construction and ones for shapes or colours were rare. Most variation in the ‘sources of play materials’ subscale came from home-made toys, which 48% of the children possessed. Over two-thirds (69.9%) of the children had ‘toys for moving around’, which were generally balls. The drawing materials tended to be pencils, and only 21% of the children had a picture book. Most (87.3%) homes had some ‘household books’ but they were mainly school books, which were distributed by the Government free of charge whereas 16% had magazines or newspapers.

**Table 2.** Frequency distribution of FCI items (n=801)

FCI subscale	%
Household books	
None	12.7
1–2	11.4
3–5	16.6
≥6	59.3
Magazines or newspapers in household	
None	84.3
1–2	1.7
3–5	3.5
≥6	10.5
Sources of play materials	
Household objects	98.0
Things from outside	99.3
Toys bought from store	84.8
Home-made toys	47.6
Mean±SD	3.3±0.7
Varieties of play materials	
Things which make/play music	16.2
Things for drawing/writing	63.0
Picture books for children (not school-books)	20.5
Things meant for stacking, con structing, building (blocks)	0.9
Things for moving around (balls, bats, etc.)	69.9
Toys for learning shapes and colours	0.4
Things for pretending (dolls, tea-set, etc.)	44.6
Mean±SD	2.1±1.4
Play activities	
Read books or look at picture-books with child	29.6
Tell stories to child	17.2
Sing songs with child	34.2
Take child outside home place	93.8
Play with the child with toys	36.6
Spend time with child in naming things, counting, drawing	62.0
Mean±SD	2.7±1.6

FCI=Family care indicator;

SD=Standard deviation

### Short- and long-term reliability

#### Short-term

To assess short-term test-retest reliability, the FCI questionnaire was repeated 7–14 days later among 40 mothers. The items that were observed (‘household books’, ‘magazines’, ‘varieties’ and ‘sources’ of play materials) were highly reliable (intraclass correlations r>0.85, p<0.001) whereas ‘play activities’ was only moderately reliable (r=0.64, p<0.001).

#### Long-term

In the 129 mothers who were given the FCI twice when their children were aged 12 and 18 months, the mean (SD) scores of ‘varieties of play materials’ increased significantly from 1.3 (1.0) at 12 months to 2.3 (1.3) at 18 months (p<0.001) but there was no significant difference in the other subscales. ‘Household books’, ‘magazines’, ‘varieties of play materials’, and ‘play activities’ were significantly but moderately correlated between 12 and 18 months (intraclass correlations r=0.62, r=0.63, r=0.52, and r=0.57 respectively, p<0.001 for all) whereas ‘sources of play materials’ showed no significant stability (r=0.09).

### Relationship of FCI with socioeconomic variables, the HOME, and child characteristics

The FCI subscales—‘household books’, ‘magazines’, ‘varieties of play materials’, ‘sources of play materials’, and ‘play activities’—were significantly related to parental education, housing, household assets, and the HOME. ‘Play activities’ and ‘varieties of play materials’ had very high correlations with the total HOME (Table 3). The number of siblings negatively correlated with ‘play activities’ and ‘magazines’ but positively with ‘household books’ probably reflecting the increase in the number of school children receiving free books from their schools. ‘Play activities’ and ‘varieties of play materials’ had low but statistically significant correlations with birthweight and gestational age. Gender was not associated with any of the subscales, except that girls tended to have fewer ‘sources of play materials’ (r=−0.10, p=0.005).

**Table 3.** Correlations between FCI subscales and socioeconomic background variables, child characteristics, total HOME score and developmental measures (n=801)

Variable	Play activities	Varieties of play materials	Sources of play materials	Household books‡	Magazines‡
Birthweight	0.11**	0.12**	NS	NS	NS
Gestational age	0.12*	0.09*	NS	0.08*	NS
BMI of mothers	0.12**	0.11**	0.01	0.11**	0.16**
Education (years) of fathers	0.31**	0.35**	0.13**	0.19**	0.36**
Education (years) of mothers	0.41**	0.40**	0.17**	0.24**	0.34**
Housing index (n=789)	0.26**	0.29**	0.10*	0.18**	0.32**
Assets (n=789)	0.35**	0.39**	0.15**	0.23**	0.36**
Income/expenditure	0.06	0.09	0.1**	0.02	0.04
Number of siblings†	−0.16**	NS	NS	0.31**	−0.14**
Total HOME	0.72**	0.73**	0.39**	0.22**	0.39**
Language					
Comprehension¶	0.44**	0.48**	0.23**	0.13*	0.22**
Expression†, ¶	0.38**	0.37**	0.18**	0.07*	0.16**
MDI‡	0.29**	0.27**	NS	NS	0.21**
PDI‡	0.19**	0.20**	0.09**	0.09*	0.18**

‡Spearmans rank correlations;

†Logged transformed;

¶Controlling for age;

*p<0.05;

**p<0.01;

BMI=Body mass index;

HOME=Home Observations for Measurement of the Environment;

MDI=Mental development index;

NS=Not significant;

PDI=Psychomotor development index

### Relationship of FCI with child development measures

After controlling for age, the correlations between the FCI subscales and the scores on the Bayley Scales and language test were examined (Table 3). ‘Play activities’, ‘varieties of play materials’, and ‘magazines’ were all significantly related to each of the four measures of child development (Table 3) whereas relationships with the ‘sources of play materials’ and ‘household books’ were less consistent. The correlations between child development outcomes and both ‘play activities’ and ‘varieties of play materials’ were similar to that observed for the total HOME score. For example, the HOME correlated with MDI (r=0.34, p<0.01), PDI (r=0.26, p<0.001), comprehension (r=0.51, p<0.001), and expression (r=0.41, p<0.001).

### Exploration of cut-off points for indicators

To explore whether the association between the FCI subscales and the Bayley MDI was linear or if there were thresholds below which child development was affected, we examined the difference in MDI scores by the number of ‘play activities’ and ‘varieties of play materials’ using ANOCOVA controlling for age. The group comprised the number of items in each scale; so, there were six levels for ‘play activities’, which had six items, seven levels for ‘varieties of play materials’, which had seven items, and nine levels for the single FCI scale, which had nine items.

We chose the two FCI subscales most strongly and consistently relating to the children's development. The MDI scores significantly differed by the number of ‘play activities’ and ‘varieties of play materials’ (group effect p<0.001 for both the scales). There were significant linear trends with each subscale (linear trend p<0.005 for both the scales), indicating that the fewer the activities or play materials the greater the risk of poor child development across the range of scores (Fig.). The difference between the children's MDI scores with the lowest and the highest number of ‘play activities’ and ‘varieties of play materials’ (excluding groups with fewer than 10 children) was very large, reaching 11 and 12 MDI points respectively.

**Fig.**
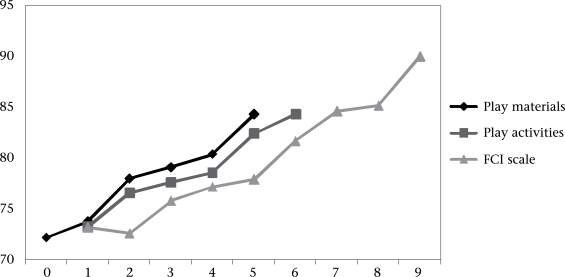
Relationship of MDI with number of ‘play materials’ and ‘play activities’ subscales and the most parsimonious FCI scale*

*Only 2 children had 6 play materials, 5 had no play activities, and 4 had ‘0’ score on the FCI scale. These points were, therefore, dropped from the plot;
FCI=Family care indicator;
MDI=Mental development index

### Independent contribution of FCI subscales to child development outcomes

Most social background and child characteristics, including birthweight and gestational age, parental education, mothers’ body mass index, housing, assets, income/expenditure ratio, and number of siblings (data not shown) significantly correlated with the child development measures. The social background variables were also related to several FCI subscales (Table 3) and were, thus, potential confounders in the relationship between FCI and child development.

To determine the independent effect of the FCI subscales on child development, we conducted series of multiple regressions of the two Bayley Scale indices and two language scores (Table 4). We entered age of the child, then offered all the potential confounders mentioned above and, finally, offered the five FCI subscales. The ‘varieties of play materials’ subscale was significant in all four regressions, and the ‘play activities’ subscale was significant in all but the regression on PDI. The ‘magazines’ subscale was significant in the regressions on MDI and PDI. Other significant covariates were household assets and income/expenditure ratio, maternal and paternal education, and gestational age. The amount of variance explained by the models ranged from 16% in PDI to 31% in language comprehension. There was some missing data from the child's height and weight but the nutritional status can also be affected by maternal care and may mediate some effect of FCI on development. We, therefore, repeated the above regressions offering the child's concurrent HAZ and WHZ scores as extra covariates in the second step. HAZ was significant in every regression (MDI: regression coefficient (B)=1.6, 95% CI 0.8–2.5, PDI: B=2.6, 95% CI 1.4–3.8; comprehension: B=0.6, 95% CI 0.2–1.1; expression: B=0.04, 95% CI 0.02–0.05, p<0.01 for all) whereas WHZ was significant only in the regression on PDI (B=2.3, 95% CI 1.1–3.5, p<0.01). All the FCI scales that were significant in the previous regressions (Table 4) remained significant, except for ‘varieties of play materials’, which was no longer significant in the regressions on MDI and PDI.

**Table 4.** Regression coefficient (B) and 95% CI of multiple regressions of developmental outcomes at 18 months on FCI subscales controlling for socioeconomic background (n=757)

Variable B (95% CI)	MDI	PDI	Language comprehension	Language expression
Age	−2.1(−2.8, −1.5)**	−2.7 (−3.4–1.9)**	0.6 (−0.1, 1.4)	0.00 (−0.02, 0.04)
Gender	-	-	-	0.08 (0.04, 0.12)**
Assets	0.9 (0.1, 1.7)	-	0.1 (−0.3, 0.6)	-
Education (years) of fathers	-	-	0.1 (0.01, 0.3)*	0.005 (0.00, 0.01)
Education (years) of mothers	0.07 (−0.2, 0.3)	0.4 (0.2, 0.7)**	−0.03 (−0.2, 0.1)	0.003 (−0.004, 0.009)
Number of siblings	−1.0 (−1.7,-0.3)**	-		
Income/expenditure	-	-	2.0 (0.9, 3.2)**	
Gestational age	1.0 (0.5, 1.4)**	1.7 (1.1, 2.3)**	0.2 (−0.06, 0.4)	0.01 (0.00, 0.02)
Play activities	1.0 (0.3, 1.6)**	-	1.0 (0.7, 1.4)**	0.04 (0.03, 0.06)**
Varieties of play materials	0.8 (0.06, 1.5)*	1.2 (0.4, 2.0)**	1.5 (1.1, 1.9)**	0.04 (0.02, 0.06)**
Magazines and newspapers	1.2 (0.3, 2.1)*	1.7 (0.6, 2.9)**	-	-
R2	0.19	0.16	0.31	0.21
F value	22.1**	27.5**	40.6**	27.3**

*p<0.05;

**p<0.01

Step 1: Age (and sex for expression) entered. Step 2: Birthweight (g), gestational age (weeks), assets (quintiles), housing, income/expenditure deficit, number of siblings, BMI of mothers, and education (years) of mothers and fathers offered. Step 3: FCI scales (play activities, sources of play materials, varieties of play materials, household books, magazines and newspapers) offered;

CI=Confidence interval;

FCI=Family care indicator;

MDI=Mental development index;

PDI=Psychomotor development index

### Independent contribution of the most parsimonious FCI scale to child-development outcomes

The most parsimonious FCI scale made a significant independent contribution to each of the developmental outcomes (Table 5). The relationship between the score and the child's MDI is also shown in the figure.

**Table 5.** Regression coefficient (B) and 95% confidence interval (95% CI) of multiple regressions of developmental outcomes at 18 months on the most parsimonious FCI scale controlling for socioeconomic background (n=757)

Variable B (95% CI)	MDI	PDI	Language comprehension	Language expression
Age	−2.1 (−2.7, −1.5)**	−2.6 (−3.4, −1.9)**	1.0 (0.06, 1.4)	0.05 (0.03, 0.07)**
Sex	-	-	-	0.07 (0.04, 0.11)**
Assets	0.9 (0.09, 1.7)*	-	0.2 (−0.16, 0.6)	-
Education (years) of fathers	-	-	0.15 (0.03, 0.26)*	0.005 (0.000, 0.01)
Education (years) of mothers	0.08 (−0.2, 0.4)	0.4 (0.1, 0.7)**	−0.07 (−0.2, 0.07)	0.002 (−0.004, 0.008)
Number of siblings	−1.0 (−1.7, −0.3)**	-	-	-
Income/expenditure	-	-	1.7 (0.7, 2.7)**	-
Gestational age	1.0 (0.5, 1.4)**	1.7 (1.1, 2.3)**	0.3 (0.06, 0.5)*	0.01 (−0.001, 0.02)
FCI scale	1.5 (1.1, 2.0)**	1.4 (0.8, 2.0)**	1.7 (1.5, 2.0)**	0.05 (0.04, 0.06)**
R2	0.20	0.16	0.33	0.24
F value	30.4**	34.9**	67.1**	40.7**

*p<0.05;

**p<0.01

Step 1: Age (and sex for expression) entered;

Step 2: Birthweight (g), gestational age (weeks), assets (quintiles), housing, income/expenditure deficit, number of siblings, BMI of mothers, and education (years) of mothers and fathers offered. Step 3: FCI scale (including play activities: songs, stories, reading, take out; sources of play materials: toys bought from store; varieties of play materials: music, drawing and writing, pretend toys; magazines and newspapers) offered;

FCI=Family care indicator;

MDI=Mental development index;

PDI=Psychomotor development index

## DISCUSSION

In general, the FCI questionnaire was easy to administer, and the mothers readily understood it. We administered the FCI questionnaire to a large number of mothers living in extreme poverty with no or limited education. Even under these circumstances, we demonstrated adequate test-retest reliability and significant relationships between the FCI and the children's development. The hypothesis that survey-type questions could reliably and validly reflect variations in the home environment was supported by these results.

The two scales—‘play activities’ and ‘varieties of play materials’—had the strongest relationship with child development measures. There was no threshold in either scale below which the children's development deteriorated. On the contrary, the continuous linear association with the outcome measures was impressive and indicates that as the scores improved so did the children's development across the range of scores. The finding suggests that these scales could be useful in monitoring the effects of interventions in the home environment. Both the scales highly correlated with the HOME scale and were nearly as closely related to the MDI and language scores as the HOME was. Given the small number of items in the ‘play activities’ and ‘varieties of play materials’, the ability of the scales to predict child development is encouraging. An important finding was that both the scales continued to be associated with the MDI and the two language scores, even controlling for many social background variables and the children's birthweight and gestational age, and the amount of variance explained by the models was similar to those of other studies that used the HOME as a measure of home stimulation (27).

The ‘sources of play materials’ subscale was much less effective in predicting child development. There was little or no variation in responses to two items—using household objects or things outside. ‘Magazines and newspapers’ were independently associated with the children's scores on the Bayley Scales, perhaps reflecting the families’ reading habits and their knowledge of current events. In contrast, it was surprising that the number of ‘household books’ was not independently related to child development; however, it was related to the number of children in the home. Although not systematically recorded, most books appeared to be free school-books and probably did not reflect the reading habits or interests of the family. In populations where free school-books are not distributed, the item on books may be more useful in predicting child development.

The Bangladeshi Language Test was easy and quick to administer and appeared to be an effective measure of child development. It had adequate levels of reliability and correlated with measures of social background and FCI subscales. It moderately correlated with other measures of child development (Hamadani JD *et al.* Personal communication, 2010). At least within this narrow age range, it shows the promise of being a tool for the assessment of language that could be adapted to various cultures. The language scores had higher correlations with the FCI and HOME than the Bayley MDI; however, collection of data on language and stimulation in the home by the same interviewer raises the potential for bias.

The most efficient subscales to predict child development would appear to be ‘play activities’, ‘varieties of play materials’, and ‘magazines and newspapers in the home’. The most parsimonious scale included only nine items from four subscales and was generally as effective in predicting the children's development. In large surveys, we would recommend using this scale for Bangladesh. However, we suggest the continued use of all five subscales for other countries until more international data are available.

In conclusion, the FCI was easy to administer, and the mothers readily understood it. It had acceptable test-retest reliability, and several of its subscales were predictive of child development. These results provide a strong platform for developing survey-based indicators of the family environment that have relevance for child development in a poor rural environment, such as Bangladesh. Further work will be necessary to determine if these relationships could also be demonstrated in other settings.

## ACKNOWLEDGEMENTS

This study was funded by United Nations Children's Fund (UNICEF) and was part of the MINIMat project which was funded by ICDDR,B, UNICEF, Swedish International Development Cooperation Agency (Sida)'s Department for Research Cooperation (SAREC), UK Medical Research Council, Swedish Research Council, Department for International Development, Global Health Research Fund, Japan, Child Health and Nutrition Research Initiative (CHNRI), Uppsala University, and United States Agency for International Development under the Cooperative Agreement No. 388-G-00-02-00125-00. ICDDR,B acknowledges with gratitude the commitment of the donors to the Centre's research efforts. ICDDR,B also gratefully acknowledges the following donors which provide unrestricted support to the Centre's research efforts: Australian Agency for International Development (AusAID), Government of the People's Republic of Bangladesh, Canadian International Development Agency (CIDA), Embassy of the Kingdom of the Netherlands (EKN), Swedish International Development Cooperation Agency (Sida), Swiss Agency for Development and Cooperation (SDC), and Department for International Development, UK (DFID).

The authors thank all the mothers and children who participated in the study, the members of the MINIMat team, the testers (Shamima Shirazi, Musarrat Rubina Mannan, Mahfuza Rahman, Qamrun Nahar, and Zohura Parveen), and interviewers (Fatema Khatun, Shiuli Rani Das, Saleha Begum, and Nasima Akhtar). The authors are also grateful to Dr. Mohammad Abdus Salam, Dr. Kuntal Kumar Saha, and Dr. Sayeeda Huq for reviewing the earlier version of the paper.
